# Classification of Geometric Forms in Mosaics Using Deep Neural Network

**DOI:** 10.3390/jimaging7080149

**Published:** 2021-08-18

**Authors:** Mridul Ghosh, Sk Md Obaidullah, Francesco Gherardini, Maria Zdimalova

**Affiliations:** 1 Department of Computer Science, Shyampur Siddheswari Mahavidyalaya, Howrah 711312, India; mridulxyz@gmail.com; 2 Department of Computer Science & Engineering, Aliah University, Kolkata 700160, India; sk.obaidullah@aliah.ac.in; 3 Department of Engineering “Enzo Ferrari”, University of Modena and Reggio Emilia, 41121 Modena, Italy; 4 Department of Mathematics and Descriptive Geometry, Slovak University of Technology in Bratislava, 810 05 Bratislava, Slovakia; maria.zdimalova@stuba.sk

**Keywords:** deep learning algorithm, convolutional neural networks, pattern classification, image-based reconstruction, cultural heritage

## Abstract

The paper addresses an image processing problem in the field of fine arts. In particular, a deep learning-based technique to classify geometric forms of artworks, such as paintings and mosaics, is presented. We proposed and tested a convolutional neural network (CNN)-based framework that autonomously quantifies the feature map and classifies it. Convolution, pooling and dense layers are three distinct categories of levels that generate attributes from the dataset images by introducing certain specified filters. As a case study, a Roman mosaic is considered, which is digitally reconstructed by close-range photogrammetry based on standard photos. During the digital transformation from a 2D perspective view of the mosaic into an orthophoto, each photo is rectified (i.e., it is an orthogonal projection of the real photo on the plane of the mosaic). Image samples of the geometric forms, e.g., triangles, squares, circles, octagons and leaves, even if they are partially deformed, were extracted from both the original and the rectified photos and originated the dataset for testing the CNN-based approach. The proposed method has proved to be robust enough to analyze the mosaic geometric forms, with an accuracy higher than 97%. Furthermore, the performance of the proposed method was compared with standard deep learning frameworks. Due to the promising results, this method can be applied to many other pattern identification problems related to artworks.

## 1. Introduction

The application of science and engineering to the analysis of artifacts and artworks such as paintings, mosaics and statues dates back several centuries [1,2,3]. However, only over the past few decades have the analytical methods developed in the mathematical, IT and physical sciences been able to gather information from the past and contribute to the analysis, interpretation and dissemination in the fine arts. In the past, there was a historical division between science and the humanities, so the interaction between these two fields has never been natural. For example, the application of signal and image processing techniques for the analysis and restoration of artworks was a very uncommon practice. Lately, there has been a greater and growing attention and interest in processing image data of artworks for storage, transmission, representation and analysis, and an increasing number of scientists with a background in analytical and mathematical techniques has approached this field, in an interdisciplinary way. There are several ways in which image processing can find significant applications in the fields of fine arts and cultural heritage. Among them, three main areas of application can be identified: obtaining a digital version of traditional photographic reproductions, pursuing imaging diagnostics and implementing virtual restoration [1,2,4]. Obtaining the exact reproduction and explanation of an artwork was one of the first developments in the first area, which includes the process of archiving, retrieving and disseminating data and derives all the benefits from the digital format [1,2,3,4,5,6]. In the second area of imaging diagnostics, digital images are used to detect and document the state of preservation of artifacts [7], as in the case of the noninvasive techniques based on imaging in different spectral regions used for the investigation of paintings [8]. In the third area, the image processing techniques can be used as a guide to the actual restoration of fine arts (computer-guided restoration), or they can produce a digitally restored version of the artwork. In some activities, the computer is more suitable than traditional artistic tools. Examples of such activities are filtering, geometric transformation of an image, segmentation and pattern recognition. Using digital technologies, every change to the image can be seen on the screen almost in real time. Moreover, images and data can be edited, filtered and processed with minimal material costs even when complicated operations are performed, e.g., changes in colors, brightness or contrast [5,9,10,11,12]. A further development consists of applying computer vision, an area of artificial intelligence, to recognize patterns of the historical art heritage [6,13].

**Figure 1:**
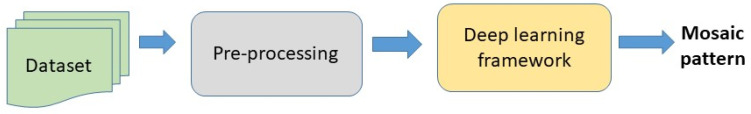
The workflow of the proposed method.

In this scenario, this paper presents a method to perform the recognition of geometrical patterns in fine arts, thanks to image processing techniques. In particular, we developed and tested a deep learning-based framework to classify the geometric forms and patterns of floor mosaics, which consist of an arrangement of tiles usually characterized by jagged and undefined boundaries or surface irregularities. The workflow of the proposed method is shown in Figure 1.

The paper is organized as follows: In Section 2, we introduce methods of image processing applied to fine arts, involving machine learning and deep learning-based techniques. Section 3 describes the proposed method based on deep neural networks. Section 4 introduces the case study. Section 5 presents the experiments resulting from the application of the deep neural network framework to the dataset and the results achieved. In Section 6, some final remarks and open questions close the paper.

## 2. Related Work

This section proposes a literature survey dealing with various methods of image processing applied to fine arts, involving machine learning and deep learning-based techniques. In [14,15,16,17,18], image processing techniques for art investigation are applied to the detection of defects and cracks, as well as to the removal of defects and canvas from high-resolution acquisition of paintings. Examples of these kinds of methods include the use of sparse representations and the removal of cradling artifacts in X-ray images of panel paintings [15] and the automated crack detection using the Ghent Altarpiece [16], employed as guidance during its ongoing restoration.

Various methods of automatic image segmentation are used in the literature aiming at identifying regions in an image and labeling them as different classes. The main applications are pattern recognition for classifying paintings [19,20,21,22,23] or the authentication of fine arts (e.g., of paintings) [24]. These image segmentation methods include the following: The thresholding methods transform a grey-scale image into a binary image, where the algorithm evaluates the differences among neighboring pixels to find object boundaries [25,26,27]. The region growing methods are based on an expansion of an object detected inside of an object [28,29] by selecting object seed pixels (inside an area to be detected) and then searching for neighboring pixels with similar intensities to the object seed pixels. In the level sets, the algorithm will converge at the boundary of the object where the differences are the highest. In the graph-cut method [30,31,32], firstly proposed by Wu and Leahy [30], each image is represented as a graph of nodes: each node corresponds to an image pixel, and links connecting the nodes are called edges; a pathway is constructed connecting all the edges to travel across the graph.

Aggregation methods are important as well for image resampling [33] or denoising [34]: When an appropriate scale or resolution is determined, the next step is to obtain the corresponding images. In the case of low scale or resolution, resampling techniques are often used to interpolate an image into a desired resolution, and aggregation is a particular resampling technique widely practiced for “up-scaling” image data from high resolution to low resolution [33].

This paper particularly focuses on deep learning [35,36], which is a kind of machine learning that uses several levels of neurons with complicated architectures or nonlinear changes to represent greater interpretations of information. With the growing volume of information and computing power, neural systems having increasingly sophisticated architecture have been of great interest and are used in a variety of disciplines. Some examples of applications in image processing and in fine arts are as follows: Image segmentation using a neural network has recently been used as a very strong tool for image processing [22,37]; recently, even convolutional neural networks have been applied to paintings [38]. In [39], a novel deep learning framework is developed to retrieve similar architectural floor plan layouts from a repository, analyzing the effect of individual deep convolutional neural network layers for the floor plan retrieval task. In [40] the results of a novel method for building structure extraction in urbanized aerial images are presented. Most of the methods are based on CNN. Similarly, in [41], the use of deep neural networks for object detection in floor plan images is investigated, evaluating the use of object detection architectures to recognize furniture objects, doors and windows in floor plans.

Gomez-Rios et al. [42] classified the textures of underwater coral patterns based on a CNN-based transfer learning-based approach. To work on diverse data and evaluate the performance of the proposed approach, they used data augmentation. The adoption of a deep neural network can significantly improve phase demodulation efficiency from a singular fringe sequence [43]. Their system was developed to anticipate several subsequent outcomes that may be used to calculate an incoming fringe pattern cycle. They collected fringe pictures of diverse situations to produce training input while the systems are being trained. The neural network blindly took only one input fringe sequence and produced the associated estimations of such transitional outcomes at great accuracy. Sandelin [44] proposed a Mask R-CNN-based technique for floor plan pictures and segmented the walls, windows, chambers and doors. This method showed good performance even in noisy images. Vilnrotter et al. [45] proposed a technique to generate appropriate naturalistic texture characteristics. The fundamental method of edge characteristics to determine an initial, incomplete identification of the components was discussed. The graphic components were extracted using such characterization. The components were classified into types and topological connections with them. The formulations were proven to be beneficial for texture identification and recurrent pattern restoration.

With a particular focus on mosaics, most of the related computer applications deal with their digital reconstruction using image-based techniques (i.e., photogrammetry) for documentation and analysis [46,47,48,49]. Besides, literature presents a few examples of image processing applications: In [50], a registration method in the framework of a restoration process of a medieval mosaic to compare a historical black and white photograph with a current digital one is presented. In [51], an algorithm that exploits deep learning and image segmentation techniques is presented to obtain a digital (vector) representation of a mosaic. In [52], the restoration of historical photographs of an ancient mosaic (by removing noise, deburring the image and increasing the contrast) and then the removal of geometrical difference between images by means of the multimodal registration using mutual information is presented; the final identification of differences between the photos indicates the changes in the mosaic during the centuries. In [53], Falomir et al. presented a mathematical method for calculating a likeness score among qualitative assessments of item structure, color and dimension in digitized pictures. The closeness scores calculated are dependent on compositional cluster maps or intermediate distances, as per the specification of the subjective characteristics. The outcome using prior techniques was enhanced by using an estimated identification process among item characteristics of a tile mosaic assembly.

## 3. Proposed Method

In this paper, we propose a deep learning-based framework to classify the forms of fine arts, such as paintings and mosaics. The algorithm is able to classify the geometrical forms constituting the patterns, even if they are partially deformed. This deep learning [54] is a type of machine learning that eliminates the need for manual processing of features. Images are immediately fed into this system, and the final categorization is returned. Due to its high capacity to cope with geographically dispersed input, the convolutional neural network (CNN) [55] is the most efficient and frequently utilized.

**Figure 2:**
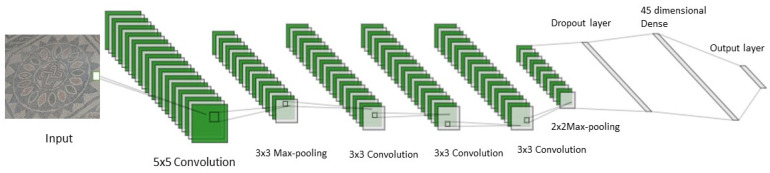
The proposed CNN architecture.

In this study, we used a CNN-based framework that autonomously quantifies the feature map and classifies it. To the best of our knowledge, there is no literature on the use of CNN for the identification of floor mosaic patterns to date. Convolution, pooling and dense layers are three distinct categories of levels found in CNN. The convolution levels generate attributes from the incoming images by introducing certain specified filters. The generated feature vector is passed through a pooling layer to reduce the spatial size of the feature map. As a result, the network parameter count and computational cost are reduced. The dense level receives all the outputs from the preceding level and delivers one output to the following level from every neuron. The proposed CNN framework can be described as CPCCCPDD architecture, where C, P and D represent convolution, pooling and dense, respectively. The input image is fed to the first convolutional layer, which consists of 32 filters having size 5 × 5. This convolutional layer is followed by a max-pool layer with filter size 3 × 3. Then three convolutional layers having 16 filters of size 3 × 3 each are fed in series. This is followed by another max-pool layer with filter size 2 × 2. There are two dense layers used in the proposed CNN framework: one is 45-dimensional dense and the second is 5-dimensional (output layer). The proposed CNN framework is depicted in Figure 2.

The number of pixels shifted across the incoming tensor is referred to as the stride. If the stride is set to 1, the filters/masks are moved one element at a time. If it is set to 2, then the mask will be shifted by two elements, and so on. Here, for both the convolution and pooling layers, the stride value of 1 is considered throughout the experiment. The dropout value of 0.5 was taken. The dropout helps to reduce the overfitting problem in the network. Before feeding to the dense layer, a batch normalization strategy is used to speed up the training process. The learning rate is taken as 0.001. The ‘Adam’ optimizer and ‘cross-entropy loss function’ are deployed in the proposed framework. In the convolutional layers and the first dense layer, the rectified linear unit (ReLU) activation function is used, which can be formularized as:

(1) f(n)=max(0,n)

where *n* is the input to a neuron.

In the output layer, the activation function named ‘Softmax’ is used, which is provided in Equation (2).

(2) $$\sigma {\left(y\right)}_{i}=\frac{e^{y_{i}}}{\sum_{l=1}^{L}e^{y_{l}}}\;$$

where y is the ith input vector of length l.

The number of parameters used in the CNN architecture is presented in Table 1. The total number of trainable parameters used is 617,491.

## 4. Case Study

**Figure 3:**
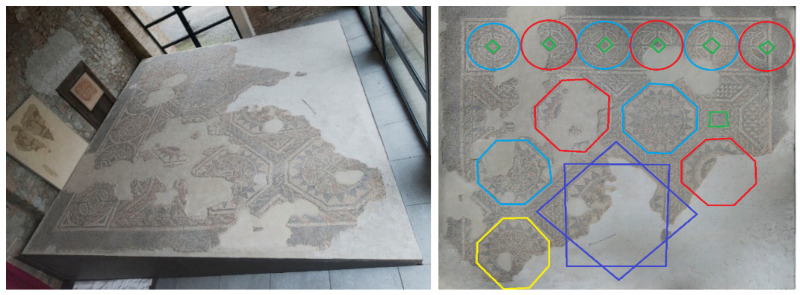
**Left**: The final location at the “Casa Natale Giuseppe Graziosi” in Savignano sul Panaro (Modena, Italy) (photo credits: Marianna Grandi, Italy). **Right**: Auxiliary geometric elements built on the orthophoto of the Roman mosaic, to highlight the geometric forms and their arrangement.

The deep learning (CNN) framework was applied and tested on a Roman mosaic discovered in Savignano sul Panaro, near the city of Modena (Italy), in 1897 during an archaeological excavation. This floor mosaic belongs to the ruins of a large late Roman building dated to the 5th century A.D. [56]. It originally measured about 6.90 m × 4.50 m, but less than half of its original surface is preserved. The Roman mosaic was removed for restoration and is now conserved in the birthplace house of the painter Giuseppe Graziosi (Savignano sul Panaro), who first documented its existence in 1897 (Figure 3, left).

The mosaic pattern is described in [57]. Its decorations present polychrome stone and terracotta tiles combined with emerald green and ruby red glass tiles. The mosaic shows a geometrical pattern of (originally) eight octagonal elements arranged around a larger central one, which consists of an eight-pointed star, formed by two superimposed squares to form a central octagon with irregular sides (in purple, in Figure 3, right). The central octagon has a circular motif with a white background containing a laurel wreath and, presumably, a figured center. The vertices of the star originate eight octagons, smaller in size, arranged in pairs of two on each side (in red, blue and yellow, in Figure 3, right), containing geometric and stylized plants that alternate with Solomon’s knots. The external octagons are only partially preserved, but all of them have internal circular motifs, with a border of pointed triangles in black on white. The space between the octagons and the side walls is filled with different polygonal and triangular forms. At the top, six circles (five full circles and one half-circle) alternate intertwined motifs with a red and black background, surrounding a central square.

A close-range photogrammetric model of the Roman mosaic is developed by means of 115 photos (standard compact camera Nikon P310 (Nikon, Tokyo, Japan), 16.1MP CMOS sensor, sensor size: 1/2.3” (~6.16 mm × 4.62 mm), max. image resolution 4608 × 3456) thanks to Agisoft Metashape Professional (Version 1.6.3). In this software, the 3D model is also scaled to its natural size using as references the sides of the inclined support of the mosaic (see Figure 3, left), whose dimensions are known. The final model consists of a detailed textured 3D model of the mosaic, which shows the arrangements of the tiles, their edges and some planar issues due to its state of conservation, as well as the geometric forms and their arrangements.

The 3D model supported the generation of images showing the mosaic geometric forms in two ways: Firstly, from the 3D model, the Agisoft Metashape Pro software developed an orthophoto, which is a computer-generated image of the whole artifact that has been corrected for any geometric distortions. In particular, it is obtained as a parallel projection of the view of a photogrammetric textured model taken along a predetermined plane [58]. During the transformation from a 2D perspective view into an orthophoto, each photo is rectified (i.e., it is an orthogonal projection of the real photo on the mosaic plane); therefore, it is no longer deformed by perspective. Conversely, the “real” photo is influenced by perspective, as seen by the human eye. Therefore, we obtained a set of 115 photographic images corrected and rectified, from which we could extract the images of geometrical forms to be classified by the deep learning algorithm.

**Figure 4:**
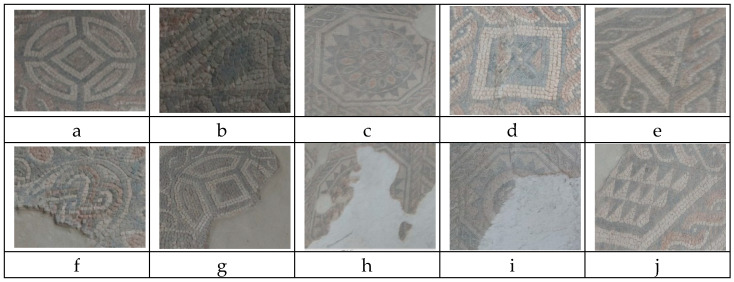
Some image samples of the mosaic forms: (**a**) circle, (**b**) leaf, (**c**) octagon, (**d**) square, (**e**) triangle; incomplete geometric forms from (**f**–**j**).

Secondly, from the 3D model, we extracted and isolated additional image samples depicting each of the geometric forms to be analyzed. By simply rotating, translating and zooming the 3D models, we obtained images of the same geometric form with multiple spatial orientations and, therefore, with multiple distortions. Some of these images are shown in Figure 4.

## 5. Experiments

### 5.1. Dataset

In this work, a dataset of images of the geometric forms of the floor mosaic was developed. Five different mosaic forms (i.e., tile patterns) were considered in this set: circles, triangles, leaves, octagons and squares.

The dataset contains 407 mosaic images, including 103 images of circles, 79 of octagons, 71 of squares, 137 of triangles and 17 of leaves. Figure 4 shows the mosaic image samples from the developed dataset, in which the mosaic tiles are arranged in patterns originating geometric forms. A circle-shaped motif of the mosaic texture is presented in Figure 4a. Similarly, (b) shows a leaf-shaped mosaic, (c) shows an octagon-shaped mosaic, (d) shows a square-shaped mosaic and (e) shows a triangle-shaped mosaic. The dataset contains images of different size such as 540 × 244, 352 × 566, 737 × 535, 869 × 760 and 1535 × 735. Since the image sizes were different, we normalized the height and width and set the size of 200 × 200 before feeding to the deep learning-based framework. The images were captured in low lighting conditions. In addition, some of the images show forms that are not completely observable. In the second row (f–j) of Figure 4, the incomplete forms of the mosaic are shown. Some incomplete circular forms are shown as semicircles in (f) and (g), and inside the circle, there is a pattern of squares (g). The remaining parts of octagonal mosaic motifs are shown in (h) and (i). In (j), there are many triangle-shaped motifs within a large square, whose actual patterns are difficult to identify. The correct identification of the mosaic forms in the dataset is complicated as the data suffer from incomplete structure, poor light condition, blurriness and low volume of data.

### 5.2. Evaluation Protocol

We used an n-fold cross-validation technique to test the efficiency of our system. In this cross-validation approach, the entire dataset was divided into n parts: training set and test set. The test set is considered as one of the *n* parts, whereas the rest (*n* − 1) are considered as the training set. In the next iteration, out of (*n* − 1) sets, one of the sets is considered as a test set (different from before), and the remaining (*n* − 1) parts are considered as the training set, and so on. This process is repeated n times. Various metrics such as accuracy, precision, recall and F-score, used to assess the effectiveness of the system, are computed as:

(3) Accuracy=((tp+tn)/(tp+fp+fn+tn))

*Precision* = *tp*/(*tp* + *fp*) (4)

*Recall* = *tp*/(*tp* + *fn*) (5)

*F-score* = (2 * *Precision* * *Recall*)/(*Precision* + *Recall*) (6)

where the true positive, false positive, false negative and true negative parameters are represented by *tp*, *fp*, *fn* and *tn*, respectively.

### 5.3. Results and Analysis

Table 2 presents the performance metrics obtained with a batch size equal to 100 and for 100 epochs. It shows that the highest accuracy of 93.61% was obtained for the 10-fold cross-validation. If the number of folds increases, the accuracy decreases.

With the 10-fold cross-validation and the batch size equal to 100, the performance of the system was analyzed by changing the number of epochs. Table 3 shows the results of the performance considering from 200 to 700 epochs with intervals of 100 epochs. It shows that, at 500 epochs, the highest values of accuracy (97.05%), recall (0.9658) and F-score (0.9651) were obtained.

Further experimentation was carried out by increasing the batch size from 50 to 250 with 50 batch intervals, keeping the 10-fold cross-validation and 500 epochs. The performance metrics are presented in Table 4, which shows that increasing the batch size did not improve the performance. The same accuracy was obtained for the batch sizes equal to 50 and 100, but higher precision and F-score were found for the batch size equal to 50.

The confusion matrix (in Table 5) was explored for the 10-fold cross-validation, a batch size equal to 50 and 500 epochs.

The confusion matrix shows that the triangle patterns present the highest accuracy (98.54%), followed by the octagons (97.46%), the circles (97.08%), the squares (94.36%) and the leaves (94.11%). The errors in identification were generated because of poor illumination, noise, blurriness and improper/incomplete geometry of the floor mosaic patterns.

### 5.4. Comparison

The performance of the system was compared to standard CNN architectures. Here, four different architectures were considered, namely VGG19 [59], MobileNetV2 [60], ResNet50 [61] and InceptionV3 [62]. VGG19, MobileNetV2, ResNet50 and InceptionV3 networks are 19, 53, 50 and 48 layers deep, while the proposed network consists of only nine layers. Instead of applying deep networks, the proposed framework gives us better performance. The comparison results are shown in Table 6.

## 6. Discussion and Conclusions

This paper presents a framework for geometric form analysis based on images extracted from a close-range photogrammetric model of an artifact (floor mosaic) and deep learning (CNN) algorithm. From the digital model of the mosaic, an orthophoto was obtained, which the photogrammetric software generated by rectifying the photos used in photogrammetry. Therefore, two sets of photos were collected in a dataset: the original photos, affected by perspective, useful for obtaining images of the deformed geometric forms of the mosaic and, on the other hand, the rectified version of the same photos with the geometric forms projected on the floor plane and so not deformed. Moreover, additional images can be obtained by simply rotating, translating and zooming the 3D model of the mosaic, generating other images with geometric forms differently deformed.

The deep learning algorithm analyzed the entire dataset consisting of 407 (normalized) images, in particular, 103 images of circles, 79 images of octagons, 71 images of squares, 137 images of triangles and 17 images of leaves. The geometric forms in the mosaic are made by arrangements of tiles, which caused jagged contours and irregularities in the geometric forms to be analyzed; moreover, there were cracks and improper/incomplete geometry of the mosaic elements, which were sometimes due to unevenness in the ground or the elements having been destroyed in the past. Moreover, some of the photos showing the mosaic forms present noise and blurs, sometimes due to poor illumination.

Despite all these defects, the algorithm is able to identify and classify more than 94% of the forms in each category, and the method has proved to be robust enough to analyze the mosaic geometric forms chosen as a case study. Furthermore, the performance of the proposed method was compared with standard deep architectures that deployed a larger number of convolutions and pooling layers than the proposed method. Instead, we achieved good accuracy using the proposed lightweight architecture.

Concerning the selected case study, the proposed method has proved to be capable of extracting and classifying data from this kind of artwork. The dataset consists of various images related to five geometric forms that are repeated in the mosaic using different arrangements of tiles, colors and orientation, usually incomplete or separated by diameters, diagonals or simply by including smaller geometric forms in larger ones. Despite all these differences among the same kinds of geometric forms, the CNN architecture has proven to be capable of classifying the five geometric forms with high accuracy; therefore, we confidentially believe that it can be easily generalized to other mosaics with similar forms and patterns. As it was not possible to test it as part of this research activity, testing the CNN algorithm with other mosaics will be planned as future work.

Additional future works will consist in the analysis of mosaics and other artworks that are not flat but 3D-shaped in space, such as curved walls, domes and vaults. In addition, the method can originate a software tool for processing and analyzing fine arts data in a more automated way.

## Figures and Tables

**Table 1:** Number of parameters used in the various levels of the CNN architecture for the presented design.

Layer	Dimension	#Parameters
Convolution 1	196 × 196 × 32	2432
Max-pool	3 × 3	-
Convolution 2	63 × 63 × 16	4624
Convolution 3	61 × 61 × 16	2320
Convolution 4	59 × 59 × 16	2320
Max-pool	2 × 2	-
Dense 1	45	605,565
Dense 2	5	230
Total		617,491

**Table 2:** The performance of the CNN architecture in different folds of cross-validation with a batch size equal to 100 and for 100 epochs.

#Fold	Accuracy	Precision	Recall	F-Score
5	91.40	0.9348	0.8912	0.9100
7	89.68	0.9108	0.8475	0.8714
10	93.61	0.9529	0.9236	0.9367
12	89.19	0.9159	0.8879	0.8960

**Table 3:** The performance evaluation by changing the number of epochs with the 10-fold cross-validation and a batch size equal to 100.

Epoch	Accuracy	Precision	Recall	F-Score
200	0.941	0.9563	0.9252	0.9395
300	96.81	0.9742	0.9599	0.9667
400	95.82	0.9658	0.9505	0.9578
500	97.05	0.9645	0.9658	0.9651
600	93.37	0.9459	0.9189	0.9313
700	91.89	0.947	0.9067	0.9246

**Table 4:** For 500 epochs and 10-fold cross-validation, the metrics were calculated by increasing the batch size from 50 to 250.

Batch	Accuracy	Precision	Recall	F-Score
50	97.05	0.9760	0.9632	0.9693
100	97.05	0.9645	0.9658	0.9651
150	93.61	0.9472	0.9253	0.9354
200	90.37	0.9021	0.8913	0.8966
250	92.87	0.9438	0.9002	0.9184

**Table 5:** The confusion matrix of the accuracy corresponding to the five floor mosaic patterns.

	Circles	Leaves	Octagons	Squares	Triangles
**Circles**	97.08	0	0	0.019	0.009
**Leaves**	0	94.11	0	0	0.058
**Octagons**	0.012	0	97.46	0	0.012
**Squares**	0.056	0	0	94.33	0
**Triangles**	0.007	0	0.007	0	98.54

**Table 6:** Comparison of the proposed framework with standard CNN-based networks.

Network	Accuracy (%)	Precision	Recall	F-Score
VGG19	93.90	0.9409	0.9278	0.9343
MobileNetV2	89.78	0.9056	0.8860	0.8956
ResNet50	84.67	0.8478	0.8408	0.8442
InceptionV3	78.55	0.7720	0.7803	0.7761
**Proposed**	**97.05**	**0.9645**	**0.9658**	**0.9651**

## References

[1] Barni M., Pelagotti A., Piva A. (2005). Image processing for the Analyses and Conversation of Paintings: Opportunity and challenges. IEEE Signal Process. Mag..

[2] Cornelis B. (2014). Image processing for art Investigation. Electron. Lett. Comput. Image Anal..

[3] Johnson C.R., Hendriks E.J., Berezhony I., Brevdo E., Huges S.M., Daubechies I., Li J., Postma E., Wang J.Z. (2008). Image processing for artist identification, Computerizes Analysis of Vincent van Gogh. IEEE Signal Process. Mag..

[4] Bartolini F., Cappellini V., Del Mastio A., Piva A. (2003). Applications of image processing technologies to fine arts. Opt. Metrol. Arts Multimed..

[5] Berezhnoy I.E., Postma E.O., van den Herik H.J. (2005). Computerized visual analysis of paintings. Proc. Int. Conf. Assoc. Hist. Comput..

[6] Teixiera G.N., Feitosa R.Q., Paciornik S. (2002). Pattern Recognition Applied in Fine Art Authentication.

[7] Amura A., Aldini A., Pagnotta S., Salerno E., Tonazzini A., Triolo P. (2021). Analysis of Diagnostic Images of Artworks and Feature Extraction: Design of a Methodology. J. Imaging.

[8] Daffara C., Ambrosini D., Di Biase R., Fontana R., Paoletti D., Pezzati L., Rossi S. Imaging data integration for painting diagnostics. Proceedings of the O3A: Optics for Arts, Architecture, and Archaeology II.

[9] Cappellini V., Barni M., Corsini M., Rosa A.D., Piva A. (2003). Artshop: An art-oriented image processing tool for cultural heritage applications. J. Visual. Comput. Animat..

[10] Milidiu R., Renteria R. (1998). Projeto Pincelada.

[11] Pei S.-C., Zeng Y.-C., Chang C.-H. (2004). Virtual Restoration of Ancient Chinese Paintings Using Color contrast Enhancement and Lacuna Texture Synthesis.

[12] Bellavia F.V., Colombo C. Color correction for image stitching by monotone cubic spline interpolation. Proceedings of the 7th Iberian Conference on Pattern Recognition and Image Analysis.

[13] Zhang D., Islam M., Lu G. (2012). A review on automatic image annotation techniques. Pattern Recognit..

[14] Cornelis B., Dooms A., Cornelis J., Schelkens P. (2012). Digital canvas removal in paintings. Signal Process..

[15] Yin R., Dunson D., Cornelis B., Brown B., Ocon N., Daubechies I. Digital Cradle Removal in X-ray Images of Art Paintings. Proceedings of the IEEE International Conference on Image Processing.

[16] Cornelis B., Ruzic T., Gezels E., Dooms A., Pizurica A., Platisa L., Cornelis J., Martens M., De Mey M., Daubechies I. (2013). Crack detection and in painting for virtual restoration of paintings: The case of the Ghent Altarpiece. Signal Process..

[17] Cornelis B., Yang Y., Vogelstein J.T., Dooms A., Daubechies I., Dunson D. Bayesian crack detection in ultra high resolution multimodal images of paintings. Proceedings of the 18th International Conference on Digital Signal Processing (DSP).

[18] Cornelis B., Dooms A., Munteanu A., Cornelis J., Schelkens P. (2010). Experimental study of canvas characterization for paintings. Computer Vision and Image Analysis of Art.

[19] Barni M., Cappellini V., Mecocci A. The use of different metrics in vector median filtering: Application to fine arts and paintings. Proceedings of the 6th European Signal Processing Conference.

[20] Lu C.S., Chung P.C., Chen C.F. (1997). Unsupervised texture segmentation via wavelet transformation. Pattern Recognit..

[21] Chen C.C., Chen C.C. (1999). Filtering methods for texture discrimination. Pattern Recognit. Lett..

[22] Castellano G., Vessio G. (2021). Deep learning approaches to pattern extraction and recognition in paintings and drawings: An overview. Neural Comput. Appl..

[23] Castellano G., Vessio G. Deep convolutional embedding for digitized painting clustering. Proceedings of the International Conference on Pattern Recognition.

[24] Stork D.G., Huang F., Wang R.C. (2010). From Digital Imaging to Computer Image Analysis of Fine Art. Lecture Notes of the Institute for Computer Sciences, Social Informatics and Telecommunications Engineering.

[25] Basvaprasad B., Hegadi R.S. (2014). A survey on traditional and graph theoretical technique for image segmentation. Inter. J. Comput. Appl..

[26] Bazi Y., Bruzzone L., Melgani F. (2007). Image thresholding based on the EM algorithm and the generalized Gaussian distribution. Pattern Recognit..

[27] Davies E.R., Davies E.R. (2012). Chapter 4—Thresholding Techniques. Computer and Machine Vision.

[28] Callara A.L., Magliaro C., Ahluwalia A., Vanello (2020). A Smart Region-Growing Algorithm for Single-Neuron Segmentation From Confocal and 2-Photon Datasets. Front. Neuroinform..

[29] Maeda J., Ishikawa C., Novianto S., Tadehara N., Suzuki Y. Rough and accurate segmentation of natural color images using fuzzy region-growing algorithm. Proceedings of the 15th International Conference on Pattern Recognition.

[30] Peng B., Zhang L., Zhang D. (2013). A survey of graph theoretical approaches to image segmentation. Pattern Recognit..

[31] Magzhan K., Matjani H. (2013). A review and evaluations of shortes path algorithm. Int. J. Sci. Technol. Res..

[32] Yi F., Moon I. Image segmentation: A survey of graph-cut methods. Proceedings of the IEEE International Conference on Systems and Informatics.

[33] Han P., Li Z., Gong J., Li D., Shan J., Gong J. (2010). Effects of Aggregation Methods on Image Classification. Technology for Earth Obs. Geospatial.

[34] Guedj B., Rengot J., Arai K., Kapoor S., Bhatia R. (2020). Non-linear Aggregation of Filters to Improve Image Denoising. Advances in Intelligent Systems and Computing.

[35] Hao X., Zhang G., Ma S. (2020). Deep learning. Int. J. Semant. Comput..

[36] Ghosh M., Mukherjee H., Obaidullah S.M., Santosh K.C., Das N., Roy K. Identifying the presence of graphical texts in scene images using CNN. Proceedings of the 2019 International Conference on Document Analysis and Recognition.

[37] Castellano G., Vessio G. A Brief Overview of Deep Learning Approaches to Pattern Extraction and Recognition in Paintings and Drawings. Proceedings of the 25th International Conference on Pattern Recognition Workshops.

[38] Castellano G., Lella E., Vessio G. (2021). Visual link retrieval and knowledge discovery in painting datasets. Multimed. Tools Appl..

[39] Sharma D., Gupta N., Chattopadhyay C., Mehta S. Daniel: A deep architecture for automatic analysis and retrieval of building floor plans. Proceedings of the 2017 14th IAPR International Conference on Document Analysis and Recognition (ICDAR).

[40] Osuna-Coutiño J.D.J., Martinez-Carranza J. (2020). Structure extraction in urbanized aerial images from a single view using a CNN-based approach. Int. J. Remote Sens..

[41] Ziran Z., Marinai S. (2018). Object detection in floor plan images. IAPR Workshop on Artificial Neural Networks in Pattern Recognition.

[42] Gómez-Ríos A., Tabik S., Luengo J., Shihavuddin A.S.M., Krawczyk B., Herrera F. (2019). Towards highly accurate coral texture images classification using deep convolutional neural networks and data augmentation. Expert Syst. Appl..

[43] Feng S., Chen Q., Gu G., Tao T., Zhang L., Hu Y., Wei Y., Zuo C. (2019). Fringe pattern analysis using deep learning. Adv. Photonics.

[44] Sandelin F. (2019). Semantic and Instance Segmentation of Room Features in Floor Plans Using Mask R-CNN. Master’s Thesis.

[45] Vilnrotter F.M., Nevatia R., Price K.E. (1986). Structural analysis of natural textures. IEEE Transactions on Pattern Analysis and Machine Intelligence.

[46] Adami A., Fassi F., Fregonese L., Piana M. (2018). Image-Based Techniques For the Survey of Mosaics in the St Mark’s Basilica in Venice. Virtual Archaeol. Rev..

[47] Doria E., Picchio F. (2020). Techniques For Mosaics Documentation Through Photogrammetry Data Acquisition. The Byzantine Mosaics Of The Nativity Church. ISPRS Ann. Photogramm. Remote Sens. Spat. Inf. Sci..

[48] Fioretti G., Acquafredda P., Calò S., Cinelli M., Germanò G., Laera A., Moccia A. (2020). Study and Conservation of the St. Nicola’s Basilica Mosaics (Bari, Italy) by Photogrammetric Survey: Mapping of Polychrome Marbles, Decorative Patterns and Past Restorations. Stud. Conserv..

[49] Fazio L., Lo Brutto M., Dardanelli G. (2019). Survey and virtual reconstruction of ancient roman floors in an archaeological context. Int. Arch. Photogramm. Remote Sens. Spat. Inf. Sci..

[50] Zitova B., Flusser J., Łroubek F. (2004). An application of image processing in the medieval mosaic conservation. Pattern Anal. Appl..

[51] Felicetti A., Paolanti M., Zingaretti P., Pierdicca R., Malinverni E.S. (2021). Mo.Se.: Mosaic image segmentation based on deep cascading learning. Virtual Archaeol. Rev..

[52] Benyoussef L., Derrode S., Filippo. S., Sebastiano B., Giovanni G. (2017). Analysis of ancient mosaic images for dedicated applications. Digital Imaging for Cultural Heritage Preservation—Analysis, Restoration, and Reconstruction of Ancient Artworks.

[53] Falomir Z., Museros L., Gonzalez-Abril L., Velasco F. (2013). Measures of similarity between qualitative descriptions of shape, colour and size applied to mosaic assembling. J. Vis. Commun. Image Represent..

[54] Ghosh M., Mukherjee H., Obaidullah S.M., Santosh K.C., Das N., Roy K. (2021). LWSINet: A deep learning-based approach towards video script identification. Multimed. Tools Appl..

[55] Ghosh M., Roy S.S., Mukherjee H., Obaidullah S.M., Santosh K.C., Roy K. (2021). Understanding movie poster: Transfer-deep learning approach for graphic-rich text recognition. Vis. Comput..

[56] Gherardini F., Santachiara M., Leali F. (2019). Enhancing heritage fruition through 3D virtual models and augmented reality: An application to Roman artefacts. Virtual Archaeol. Rev..

[57] Santachiara M., Gherardini F., Leali F. (2018). An Augmented Reality Application for the Visualization and the Pattern Analysis of a Roman Mosaic. IOP Conference Series: Materials Science and Engineering, Kuala Lumpur, Malaysia, 13–14 August 2018.

[58] Ippolito A., Cigola M. (2017). Handbook of Research on Emerging Technologies for Digital Preservation and Information Modeling.

[59] Simonyan K., Zisserman A. (2014). Very deep convolutional networks for large-scale image recognition. arXiv.

[60] Howard A.G., Zhu M., Chen B., Kalenichenko D., Wang W., Weyand T., Andreetto M., Adam H. (2017). Mobilenets: Efficient convolutional neural networks for mobile vision applications. arXiv.

[61] He K., Zhang X., Ren S., Sun J. Deep residual learning for image recognition. Proceedings of the IEEE Conference on Computer Vision and Pattern Recognition.

[62] Szegedy C., Vanhoucke V., Ioffe S., Shlens J., Wojna Z. Rethinking the inception architecture for computer vision. Proceedings of the IEEE Conference on Computer Vision and Pattern Recognition.

